# Impact of surgically placed radiopaque markers during aortic root surgery on facilitating secondary diagnostic and therapeutic interventions

**DOI:** 10.1186/s13019-023-02365-4

**Published:** 2023-09-26

**Authors:** Emmanuel Zimmer, Maria Nucera, Clarence Pingpoh, Murat Yildiz, Paul Puiu, Florian Schoenhoff, Martin Czerny, Matthias Siepe

**Affiliations:** 1 Department of Cardiac Surgery, Cardiovascular Center, Inselspital University Hospital Bern, Bern, Switzerland; 2 Department of Cardiovascular Surgery, University Heart Center Freiburg-Bad Krozingen, Freiburg, Germany; 3 Faculty of Medicine, Albert Ludwigs University Freiburg, Freiburg, Germany; 4 Internal Medicine Clinic, University Hospital Basel, Basel, Switzerland

**Keywords:** Aortic root surgery, Radiopaque markers

## Abstract

**Background:**

Implantation of radiopaque markers during aortic root surgery might possibly facilitate upcoming coronary angiography or transcatheter aortic valve implantation. Aim of this study was to report the impact of surgically placed radiopaque markers on procedural characteristics and on angiographic outcomes.

**Methods:**

We retrospectively analyzed baseline characteristics, preoperative and postoperative data as well as procedural findings. In addition, a subgroup analysis of all patients who underwent coronary angiography after aortic root surgery was performed to report radiation time and contrast media used.

**Results:**

A total of 469 patients underwent aortic root surgery between January 2008 and April 2020. Patients were divided into two groups: group w/ markers (n = 182) and group w/o markers (n = 287). A propensity score matching was performed resulting in a total of 28 patients w/ markers and 28 patients w/o markers. Aortic cross-clamp time did not differ statistically significantly between the group w/o markers and the group w/ markers (124.0 [96.0–150.0] versus 123.0 [110.0–149.0] min, *p* = 0.09). There was no increased probability for requirement of postoperative angiography in the group w/o markers compared to the group w/ markers (11.8% versus 15.4%, *p* = 0.27). There was no statistically significant difference in the radiation time 5.5 [3–6.5] versus 5 [2.5–7.5] min, *p* = 0.62) nor in the amount of contrast media used (85 [77.5–100] versus 80 [60–90] ml, *p* = 0.07).

**Conclusions:**

Surgically placed radiopaque markers during aortic root surgery do not increase operative risk and have the potential for facilitating secondary diagnostic and therapeutic interventions.

## Background

Among the surgical options to treat aortic root aneurysm, valve-sparing aortic root replacement (VSARR), which include reimplantation and remodeling techniques, and composite valve graft (CVG) are the most commonly used strategies [1–4]. A meticulous surgical technique is necessary to obtain correct and geometric reimplantation of coronary ostia in order to achieve an optimal result. Particular attention is needed in bicuspid aortic valve where ostial geometry can be different according to the individual cuspidity when performing VSARR.

Radiopaque bypass graft markers have been shown to improve the detection of bypass grafts during subsequent coronary angiography and are associated with a lower radiation time and less consumption of contrast agent [5].

We hypothesize that marking the proximal end of the aortic graft in VSARR and marking the neo-ostia in all aortic root surgery might improve their detection during subsequent percutaneous interventions and could be reflected in a shorter examination time as well as in a reduced radiation dose and a smaller amount of contrast media used as shown in previous studies [5].

In this article, we intend to analyze safety and efficacy of surgically placed radiopaque markers in the proximal end of the aortic graft and the neo-ostia during aortic root surgery.

## Patients and methods

### Study population and design

This retrospective study analyzed patients with a history of aortic root surgery at the Department of cardiovascular surgery, University Heart Center Freiburg - Bad Krozingen, Germany, between January 2008 and April 2020. A total of 469 consecutive patients underwent aortic root surgery (VSARR and CVG) and marking was performed by surgeon’s preference. Patients were divided into two cohorts: group w/ markers (n = 182) and group w/o markers (n = 287).

We analyzed baseline characteristics, preoperative and postoperative data as well as procedural findings. In patients with secondary diagnostic and therapeutic interventions, radiation time and contrast media consumption were reported.

### Marking technique

**Fig. 1:**
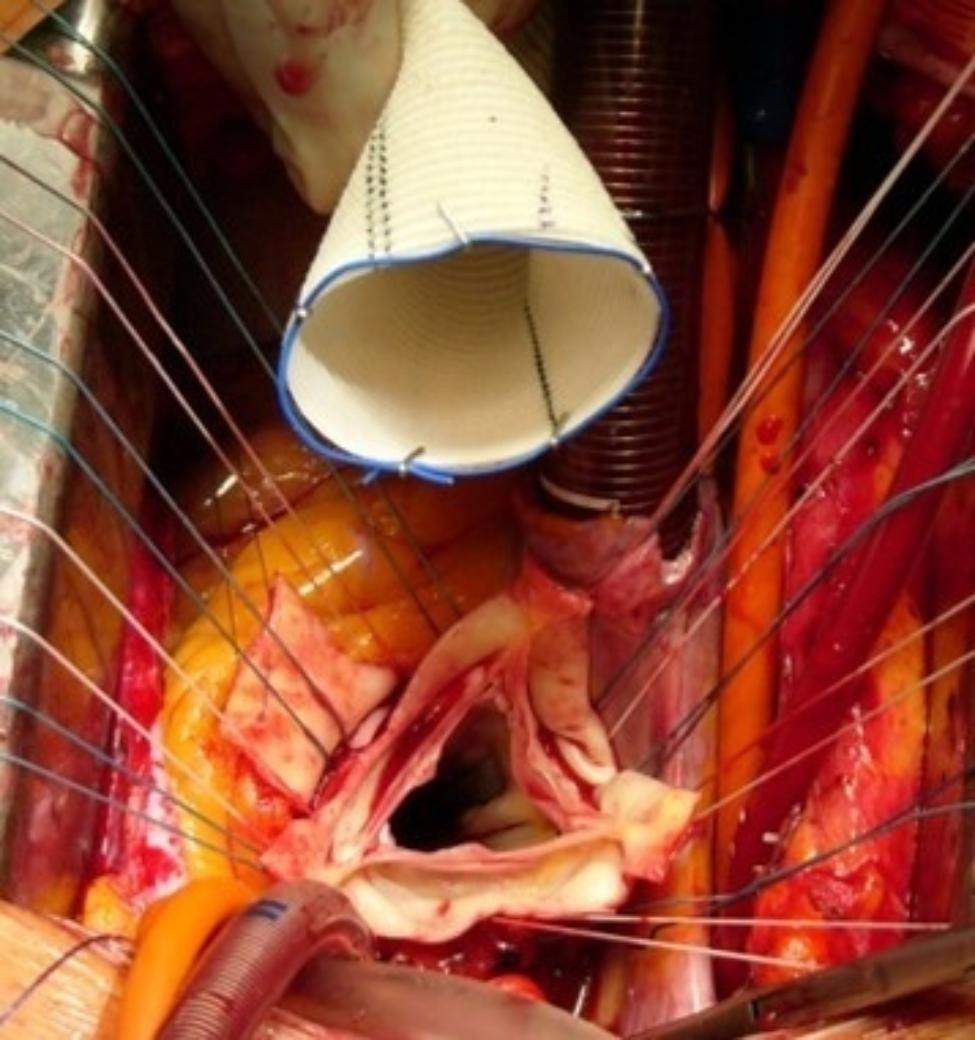
Radiopaque markers of the base of the aortic graft

**Fig. 2:**
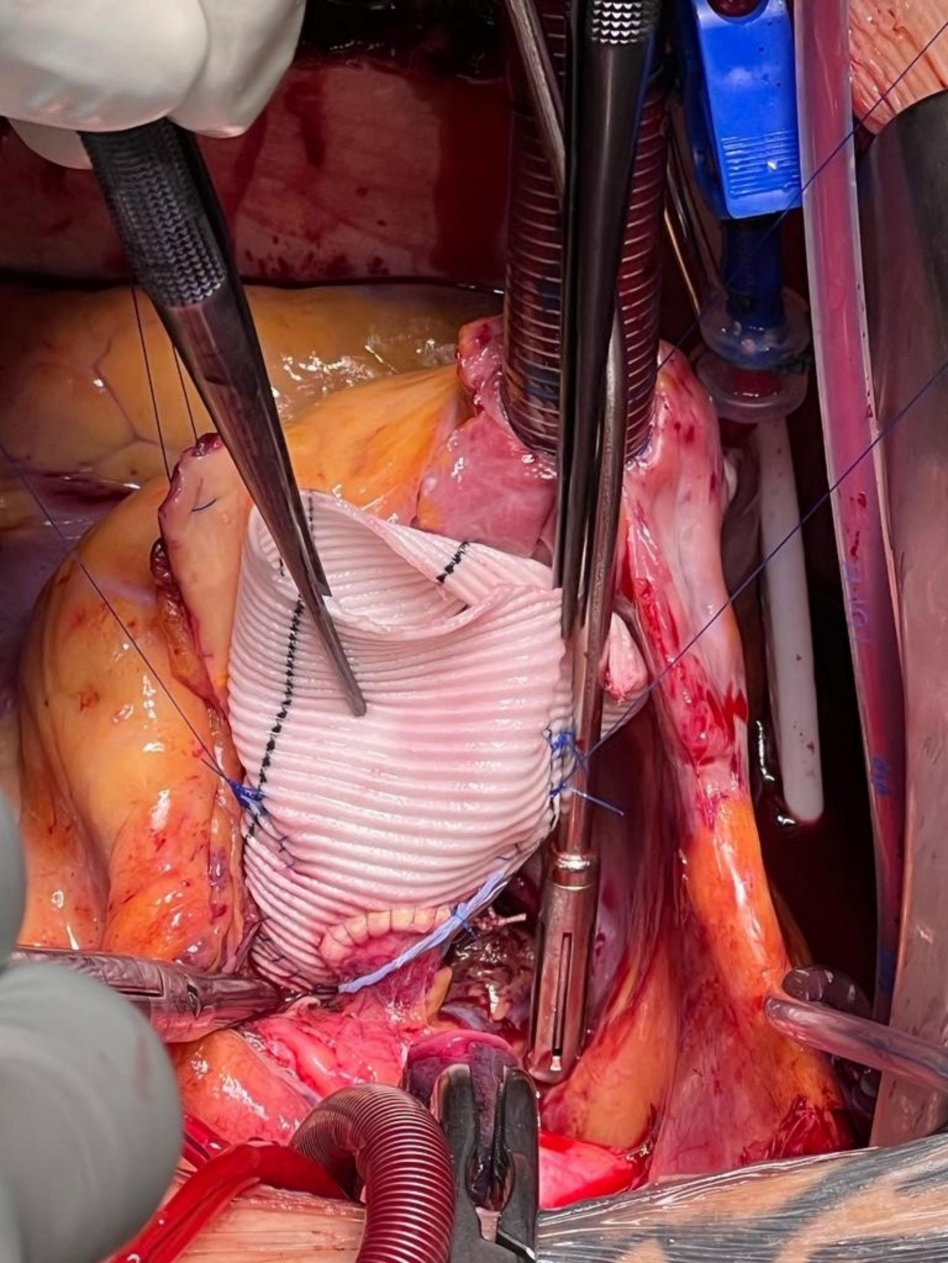
Radiopaque markers of the neo-ostia

At our institution, the marking strip of a sterile swap is used to firstly mark the proximal end of the aortic graft which is fixed with clips (Fig. 1) and secondly to mark the neo-ostia which is knotted loosely with the suture material or fixed with clips (Fig. 2). There are currently no commercially available graft markers in Europe that are authorized for this use.

### Statistical analysis

Statistical analysis was performed using R statistical software for MacOS (Stata/MP version 13.0, StataCorp, College Station, Texas) and p-values < 0.05 were considered statistically significant.

The distribution of continuous data was assessed by Shapiro-Wilk tests. Non-normally distributed continuous data are presented as median and interquartile range [IQR] and were compared using the Wilcoxon-Mann-Whitney test. Categorical and binary variables are presented as total numbers (n) and proportion in percentages (%) and were compared using the Pearson chi-square test, applying the Fisher exact test when n was less than 5.

To minimize selection bias and obtain comparable groups, we used a propensity score matching analysis. The 2 groups were matched in a 1:1 ratio with the caliper set at 0.2 of SD of the logit of the propensity score resulting in a total of 28 patients w/ markers and 28 patients w/o markers.

## Results

A total of 469 patients who underwent aortic root surgery at our institution between January 2008 and April 2020 were analyzed. Mean follow-up of the patients w/o markers was 1.98 ± 2.75 years and of the patients w/ markers 0.87 ± 1.22 years. Baseline and operative characteristics were significantly different (*p* < 0.05) between the group w/o marks and the group w/ marks: age (60.0 [52.0–69.0] versus 56.0 [46.0-65-0] years), BMI (27.0 [25.0-30-0] versus 25.5 [23.0–28.0] kg/m^2^) and intraoperative lowest body temperature (35.0 [32.0–36.0] versus 35.3 [34.8–35.9] °C). There were slightly more emergency operations in the group w/o markers (5.9% versus 2.2%, *p* = 0.06). Hospital stay was significant longer in the group w/o markers compared to the group w/ markers (15 [12–19] versus 13 [11–16] days, *p* < 0.01). However, intensive care unit stay did not differ statistically significantly between the group w/o markers compared to group w/ markers (0.98 [0.85–2.86] versus 0.95 [0.88–1.99] days, *p* = 0.97). 30-day mortality was significantly higher in the group w/o markers (9.8% versus 1.6%, *p* < 0.01). Baseline characteristics are presented in Table 1.

**Table 1:** Baseline Characteristics

	w/o markers n = 287	w/ markers n = 182	*P*
Age, y	60.0 [52.0–69.0]	56.0 [46.0–65.0]	< 0.01
Female	40 (13.9%)	30 (16.5%)	0.45
BMI, kg/m^2^	27 [25–30]	25.5 [23, 28]	0.00
Arterial hypertension	280 (97.6%)	178 (97.8%)	1.00
Diabetes mellitus	7 (2.4%)	4 (2.2%)	0.87
Creatinine, mg/dl	0.9 [0.8–1.1]	0.9 [0.8–1.1]	0.67
GFR, ml/min	84.9 [68.3–95.4]	86.5 [74.6–97.1]	0.13
NYHA			0.83
I II III IV	280 (97.6%) 3 (1.0%) 2 (0.7%) 2 (0.7%)	178 (97.8%) 3 (1.6%) 1 (0.5%) 0 (0.0%)	
LVEF, %	53 [45–60]	56 [47–61]	0.20
CAOD			0.05
No CAOD 1 vessel 2 vessels 3 vessels	214 (74.6%) 30 (10.5%) 28 (9.8%) 15 (5.2%)	142 (77.0%) 19 (10.4%) 11 (6.0%) 10 (5.5%)	
Previous cardiac surgery	6 (2.1%)	1 (0.5%)	0.18
Emergency operation	17 (5.9%)	4 (2.2%)	0.06
In-hospital stay, d	15 [12–19]	13 [11–16]	< 0.01
Intensive unit care stay, d	0.98 [0.85–2.86]	0.95 [0.88–1.99]	0.97
30-days mortality	28 (9.8%)	3 (1.6%)	< 0.01

Continuous data are presented as median [IQR] and categorical data are presented as n (%)

*BMI: body mass index, GFR: glomerular filtration rate, LVEF: left ventricular ejection fraction, CAOD: coronary artery occlusive disease*

After propensity score matching, there was no statistically significant difference. Baseline characteristics after propensity score matching are presented in Table 2.

**Table 2:** Baseline Characteristics in Propensity Score Matched Population

	w/o markers n = 28	w/ markers n = 28	*P*
Age, y	63 [54.0, 64.0]	57 [56.0, 58.0]	0.82
Female sex	4 (14%)	3 (11%)	0.69
BMI, kg/m^2^	28 [26, 30]	27 [26, 28]	0.68
Arterial hypertension	28 (100%)	28 (100%)	
Diabetes mellitus	2 (7%)	1 (4%)	0.55
Creatinin, mg/dl	0.9 [0.8, 1.1]	0.9 [0.8, 1.1]	0.67
GFR, ml/min/surface	83 [72, 94]	85 [74, 96]	0.44
NYHA			
I II III IV	28 (100%) 0 (0%) 0 (0%) 0 (0%)	28 (100%) 0 (0%) 0 (0%) 0 (0%)	
LVEF, %	53 [46, 60]	54 [47, 61]	0.44
CAOD	8 (29%)	5 (18%)	0.24
Previous cardiac surgery	0 (0%)	0 (0%)	
Emergency operation	1 (4%)	0 (0%)	0.31

Continuous data are presented as median [IQR] and categorical data are presented as n (%)

*BMI: body mass index, GFR: glomerular filtration rate, LVEF: left ventricular ejection fraction, CAOD: coronary artery occlusive disease*

### Safety of radiopaque markers

Aortic cross-clamp time did not differ statistically significantly between the group w/o markers and the group w/ markers (124.0 [96.0–150.0] versus 123.0 [110.0–149.0] min, *p* = 0.09). Perioperative myocardial infarction, characterized by an increase in troponin [6], did not differ statistically significantly between the group w/o markers and the group w/ markers (1.0 [0.6–1.7] versus 1.0 [0.7–1.6] ng/ml, *p* = 0.65). There is no significantly increased probability for requirement of postoperative angiography in the group w/o markers compared to the group w/ markers (11.8% versus 15.4%, *p* = 0.27). The need for early or late PCI did not differ statistically significantly between the group w/o markers compared to the group w/ markers (*p* = 1.00). Outcomes are presented in Table 3.

**Table 3:** Outcomes of Safety and Efficacy

	w/o markers n = 287	w/ markers n = 182	*P*
CPB time, min	157.0 [122.0-190.0]	146.0 [128.0-179.0]	0.41
Aortic cross-clamp time (min)	124.0 [96.0-150.0]	123.0 [110.0-149.0]	0.09
Troponin max. (ng/ml)	1.0 [0.6–1.7]	1.0 [0.7–1.6]	0.65
Angiography	34 (11.8%)	28 (15.4%)	0.27
PCI			
No PCI Early PCI Late PCI	282 (98.3%) 3 (1.0%) 2 (0.7%)	179 (98.4%) 2 (1.1%) 1 (0.5%)	1.00
Dose area product (cGy/cm^2^)	4024.5 [2190–5333]	3271.5 (1968.5-5003.5)	0.63
Contrast agent used (ml)	85 [70–100]	80 [60–90]	0.10
Radiation time (min)	4.5 [3–6]	5 [2.5–7.5]	0.72

Continuous data are presented as median [IQR] and categorical data are presented as n (%)

*CPB: cardiopulmonary bypass, PCI: percutaneous coronary intervention*

After propensity score matching, there was no statistically significant difference. Outcomes after propensity score matching are presented in Table 4.

**Table 4:** Outcomes of Safety and Efficacy in Propensity Score Matched Population

	w/o markers n = 28	w/ markers n = 28	*P*
CPB time, min	157.0 [122.0-190.0]	146.0 [128.0-179.0]	0.41
Aortic cross-clamp time (min)	124.0 [96.0-150.0]	123.0 [110.0-149.0]	0.09
Dose area product (cGy/cm^2^)	4124.5 [2940-5359.5]	3271.5 [1968.5-5003.5]	0.30
Contrast agent used (ml)	85 [77.5–100]	80 [60–90]	0.07
Radiation time (min)	5.5 [3-6.5]	5 [2.5–7.5]	0.62

Continuous data are presented as median [IQR] and categorical data are presented as n (%)

*CPB: cardiopulmonary bypass, PCI: percutaneous coronary intervention*

### Efficacy of radiopaque markers

**Fig. 3:**
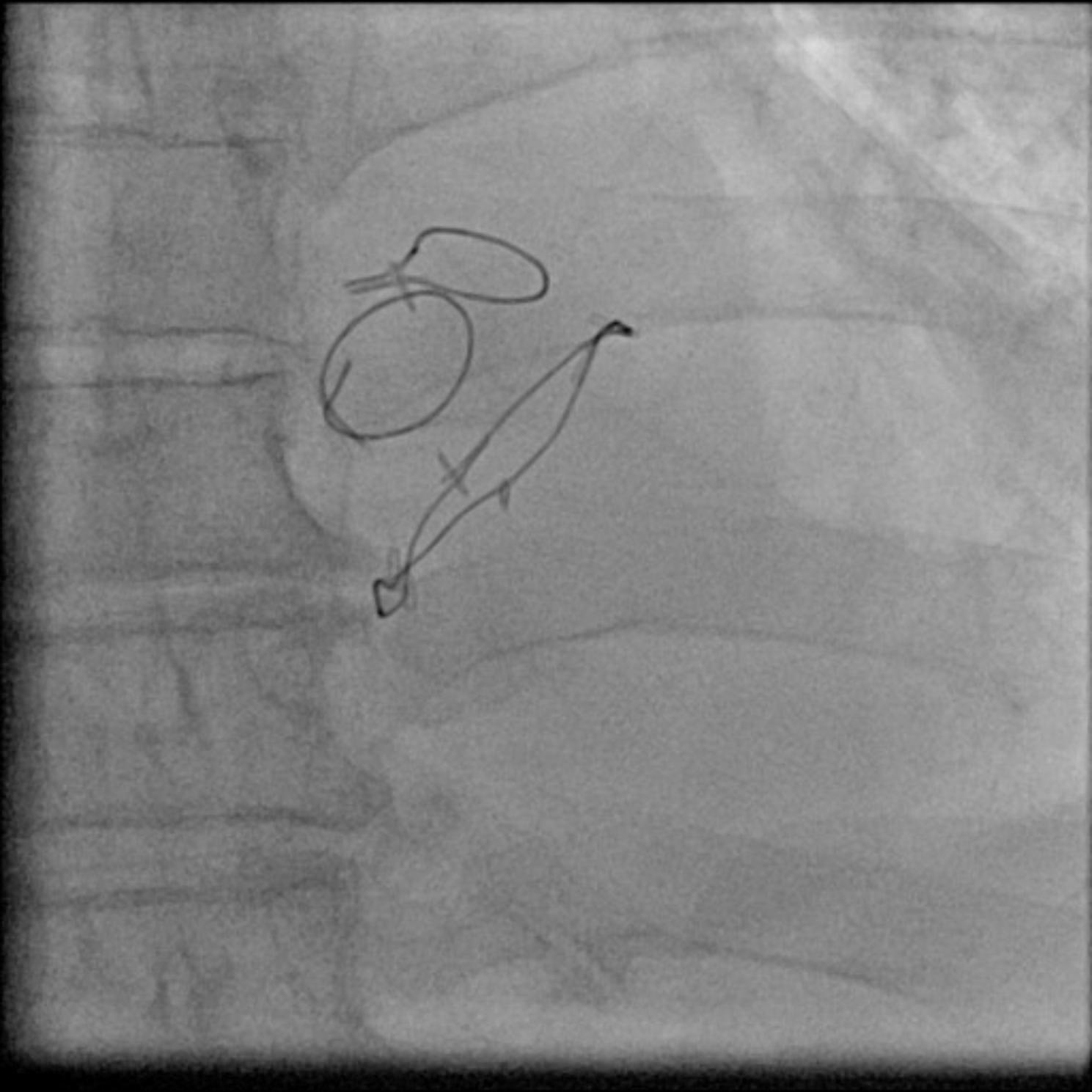
Postoperative fluoroscopy showing the different markers

The dose area product did not significantly differ in the group w/o markers compared to the group w/ markers (4024.5 [2190–5333] versus 3271.5 [1968.5–5003.5] cGy/cm^2^, *p* = 0.63). There is numerically more, but no statistically significant increased amount of contrast agent used in the group w/o markers compared to group w/ markers (85 [70–100] versus 80 [60–90] ml, *p* = 0.10). There was no statistically significant difference in the radiation time between the group w/o markers and the group w/ markers (4.5 [3–6] versus 5 [2.5–7.5] min, *p* = 0.72). Outcomes are presented in Table 3. Figure 3 shows a postoperative fluoroscopy with the different markers.

After propensity score matching, there was no statistically significant difference. Outcomes after propensity score matching are presented in Table 4.

## Discussion

Aortic root surgery especially valve-sparing aortic root replacement is still a challenging field of cardiac surgery. In the long-term follow-up, accurate localization of the neo-ostia and the proximal end of the aortic graft are crucial for further diagnosis and treatment. In order to achieve this, we have begun to regularly mark the neo-ostia during VSARR and CVG and the proximal end of the aortic graft in VSARR with radiopaque material. The advantage of the marking technique has already been demonstrated in coronary bypass surgery with improved detection of bypass grafts during subsequent coronary angiography and hence lower radiation time and less consumption of contrast agent [5].

In this retrospective study, we could show that the two groups did not differ concerning the duration of the operation and cross clamp time. Moreover, in patients requiring postoperative angiography, there was no incidence for a longer radiation time.

Furthermore, there was no increased rate of neo-ostia stenosis neither postoperatively nor in the long-term follow-up. This is illustrated by identical numbers for early and late PCI in both groups.

Total hospital stay and 30-day mortality rates are significantly lower in marked patients. It is noticeable that the marked patients are significantly younger and have significantly lower BMI whereas there were slightly more emergency operations in the group w/o markers. With regard to the other variables, both groups do not differ significantly.

Also surgical re-intervention after VSARR or CVG failure is technically challenging and associated with increased perioperative risk such as heart and/or aortic injury upon chest re-entry or the need for extensive aortic resection. Aicher et al. demonstrate that repair failure might be successfully re-repaired resulting in 92% freedom from valve replacement after 10 years [7]. However, several problems unique to this patient collective also make transcatheter aortic valve replacement (TAVR) more complex. These include the fact that aortic regurgitation is the primary mode of (homo) graft failure, there is a lack of extensive valvular leaflet calcification, and there is no clearly identifiable landing zone for a TAVR valve [8]. With the current trend in percutaneous valve implantation, these patients may be contenders for a TAVR procedure. There is only one documented case of TAVR after David operation reported by Favero et al. 8]. We did not have any cases of TAVR after valve-sparing aortic root replacement in our cohort. We believe that this is due to the follow-up period being too short. Aortic regurgitation after valve-sparing aortic root replacement mainly occurred in younger patients and these patients do not qualify for TAVR. Devices and indications might change over time which may possibly allow for safer use of the TAVR technique in these patients in the future. With the radiopaque marker, at least one of the major challenges for a post-David TAVR procedure could be solved: identifiable landing zone. However, as there were no patients requiring this procedure, we were unable to demonstrate any benefits of this marking.

### Limitation

The current study carries all limitations associated with the retrospective nature of this study, including the possibility of bias. In particular, confounding cannot be excluded. There is a selection bias that cannot be completely eliminated even with a propensity-score matching since the marking was primarily performed by two surgeons after a certain point in time. However, the amount of contrast media and the radiation time should not be affected by this variable. The low number of patients and events limits the possibility to detect relevant differences. Since the product used for marking is not a commercially available product, each clinic need to do its in-house production in agreement with the local legal bodies. Even though not observed in our experience, surgical clips used to fix the markers might result in artifacts in echo and CT. Therefore, we do not use clips for the marking of the ostia but only sutures instead. In the future we also intend to apply this method to the marking of the proximal end of the aortic graft.

## Conclusion

Surgically placed radiopaque markers during aortic root surgery do not increase operative risk and have the potential for facilitating secondary diagnostic and therapeutic interventions. The need for these secondary intervention during mid-term follow-up is extremely low due to a highly standardized and reproducible procedure. Longer follow-up is needed to substantiate this strategy.

## Data Availability

All relevant data are within the manuscript. Derived data supporting the findings of this study are available from the corresponding author on request.

## Abbreviations

CVG: composite valve graft
TAVR: transcatheter aortic valve replacement
VSARR: valve-sparing aortic root replacement
